# Peritoneal Metastatic Cancer Stem Cells of Gastric Cancer with Partial Mesenchymal-Epithelial Transition and Enhanced Invasiveness in an Intraperitoneal Transplantation Model

**DOI:** 10.1155/2020/3256538

**Published:** 2020-08-05

**Authors:** Xiao-Hai Song, Xin-Zu Chen, Xiao-Long Chen, Kai Liu, Wei-Han Zhang, Xian-Ming Mo, Jian-Kun Hu

**Affiliations:** ^1^Department of Gastrointestinal Surgery & Laboratory of Gastric Cancer, West China Hospital, Sichuan University, Chengdu, China; ^2^Laboratory of Stem Cell Biology, West China Hospital, Sichuan University, Chengdu, China

## Abstract

**Objectives:**

This preliminary study is aimed at enriching and isolating peritoneal metastatic cancer stem cells (pMCSCs) of gastric cancer and assessing their epithelial-mesenchymal transition (EMT) phenotype and invasiveness.

**Methods:**

Cancer stem cells of human gastric cancer (CSC-hGC) were previously isolated and transfected with green fluorescent protein and luciferase genes to validate the mouse model of peritoneal metastasis established via transplantation. The first and second generations ([G1] and [G2], respectively) of pMCSCs were isolated from intraperitoneally transplanted CSC-hGC (pMCSC-tGC) by spherical culture. CSC and EMT-related markers and regulators in the two generations of intraperitoneally transplanted tumors were examined by immunohistochemistry, immunofluorescence staining, and quantitative PCR. Cell mobility was examined by a transwell assay.

**Results:**

The nude mouse model of intraperitoneally transplanted CSC-hGC was successful in establishing sequential formation of peritoneal tumors and enrichment of pMCSCs. CD44 and CD54 were consistently expressed in the two generations of transplanted tumors. In vitro cell (migration) assays and immunocytofluorescence assays showed that in pMCSC-tGC^[G2]^, E-cad, Survivin, and Vimentin expression was stable; *α*-SMA expression was decreased; and OVOL2, GRHL2, and ZEB1 expression was increased. PCR analysis indicated that in pMCSC-tGC^[G2]^, the mRNA expression of E-cad, *α*-SMA, MMP9, MMP2, and Vimentin was downregulated, while that of ZEB1, OVOL2, and GRHL2 was upregulated. In vivo tumor (homing) assays and immunohistochemical assays demonstrated that in pMCSC-tGC^[G2]^, E-cad and Snail were upregulated, while *α*-SMA was downregulated. The numbers of migrated and invaded pMCSC-tGC^[G1]^ and pMCSC-tGC^[G2]^ were significantly higher than those of CSC-hGC in migration and invasion assays.

**Conclusions:**

pMCSCs might be a specific subpopulation that can be sequentially enriched by intraperitoneal transplantation. pMCSCs exhibited a tendency towards partial mesenchymal-epithelial transition, enhancing their invasiveness during homing and the formation of peritoneal tumors. However, these preliminary findings require validation in further experiments.

## 1. Introduction

Gastric cancer (GC) remains one of the most common malignancies, with high worldwide mortality [1]. In China, most GC patients are diagnosed with locally advanced or metastatic disease [2–4]. Peritoneal metastasis is found in up to 30% of GC patients at initial diagnosis and is the most common recurrence pattern after radical resection for locally advanced GC [5]. The median survival time of GC is only 3.1 months after diagnosis of peritoneal metastasis [6]. Cytoreductive surgery, systematic chemotherapy, and/or hyperthermic intraperitoneal chemotherapy are insufficient to improve the survival of these patients [7–9].

A minor subpopulation of tumor cells in a given tumor that have the potential for tumor initiation and tumor progression, called cancer stem cells (CSCs), has recently been identified [10, 11]. CSCs are characterized by their abilities for self-renewal, tumorigenesis, and differentiation [12–14]. CSCs were identified first in hematologic malignancies [15] and then in various solid tumors [11, 16]. Takaishi et al. isolated GC-initiating cells through identification of the cell surface marker CD44 [17]. We identified CSCs from the primary tumors and peripheral blood of GC patients through their expression of the surface markers CD44 and CD54 [18].

Epithelial cells exhibit clonal heterogeneity [19], which suggests the possibility of diverse clusters of initial CSCs. Therefore, it was hypothesized that a specific subpopulation, metastatic CSCs (MCSCs), might be responsible for metastasis [20]. The CSCs isolated from peripheral blood had stronger migratory and invasive abilities than those isolated from primary tumors [18]. A common feature of the tumor metastasis process is that specialized epithelial cells lose their adhesion and polarity, reorganize their cytoskeleton, and acquire a mesenchymal morphology and migration ability [21]. Moreover, compared with circulatory metastasis, peritoneal metastasis from GC might be a distinguishable pattern, especially regarding the homing of MCSCs. A cluster of peritoneal MCSCs (pMCSCs) may exist and function in GC peritoneal metastasis [22]. Therefore, we established a nude mouse model of peritoneal metastasis with CSCs of human GC (CSC-hGC) through intraperitoneal injection and aimed to enrich pMCSCs from intraperitoneally transplanted CSC-hGC (pMCSC-tGC) and characterize their stemness, tumorigenicity, and invasiveness.

## 2. Materials and Methods

### 2.1. CSC-hGC and Animals

CSC-hGC have previously been isolated from primary GC tissues, as reported elsewhere [23, 24]. The specific markers of gastric CSCs are still controversial and under discussion. In our previous study, gastric CSCs were identified and isolated through their expression of the CSC markers CD44 and CD54. Our study indicated that the cells with a CD44(+)CD54(+) phenotype had characteristics of stem cells, as confirmed by serial transplantation in vivo and serial clone formation in vitro. Informed consent was obtained from patients who provided samples, and this study was approved by the Biomedical Ethics Committees of West China Hospital.

**Figure 1:**
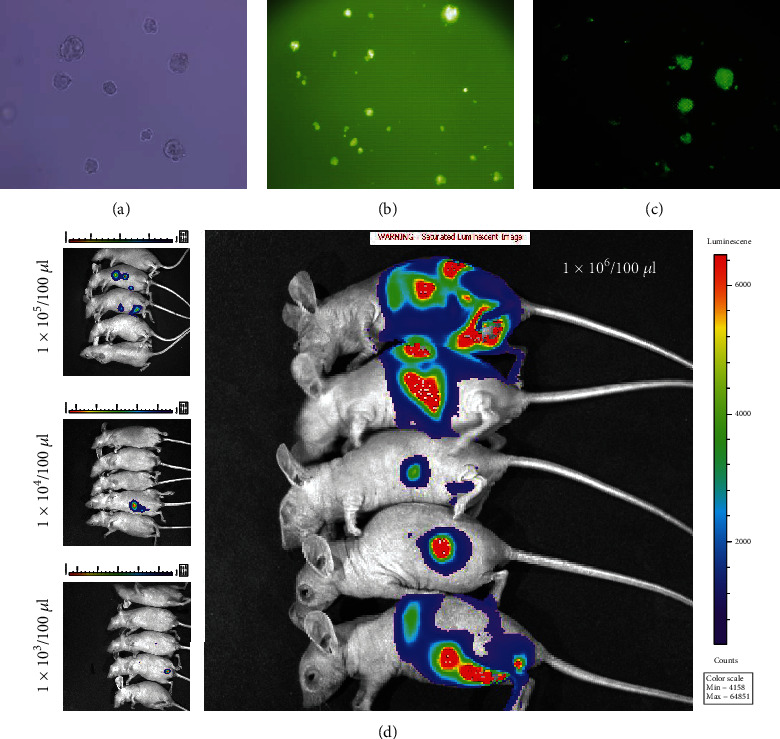
(a) The CSC-hGC sphere by subculture (×200). (b, c) The successfully transfected CSC-hGC^[GFP+LUC]^ by immunocytofluorescence (×200). (d) The bioluminescence imaging for nude mouse models injected intraperitoneally with 100 *μ*l of 1 × 10^3^, 1 × 10^4^, 1 × 10^5^, and 1 × 10^6^ CSC-hGC^[GFP+LUC]^.

CSC-hGC expressed the surface markers CD44 and CD54 and formed xenografts with a morphology similar to that of the primary tumors. The CSC-hGC used in the present experiments were CSCs isolated from a poorly differentiated gastric adenocarcinoma (id: GCSC.112) (Figure 1(a)). Four-week-old female nude mice (Da Shuo Co., Ltd., Chengdu, China) were maintained under standard housing conditions at Sichuan University. The Institutional Biomedical Ethics Committee of West China Hospital approved the study protocol, and animal experiments were conducted according to the guidelines of the local Institutional Animal Care and Use Committee.

### 2.2. Lentiviral Transduction

CSC-hGC were detached with Accutase (Sigma, U.S.), seeded in a 96-well plate at a concentration of 4 × 10^4^ cells/ml, and cultured to a confluence of between 50% and 70%. Concentrated virus expressing green fluorescent protein and luciferase (GFP+LUC) (Hanbio Biotechnology Co., Ltd., Shanghai, China) stored in a –80°C freezer was thawed on ice. Then, 100 *μ*l of serum-free medium and concentrated virus were added together to the 96-well plate. After infection for 72 hours, the fluorescence intensity of the CSC-hGC was observed under an inverted fluorescence microscope (Olympus, Japan). The CSC-hGC^[GFP+LUC]^ with the strongest fluorescence intensity and most robust growth were selected. Next, CSC-hGC^[GFP+LUC]^ with stable expression of GFP+LUC were obtained by puromycin selection.

### 2.3. Animal Model of Peritoneal Metastasis

Five mice were assigned to each group for separate experiments. Each mouse was injected with 1 × 10^3^, 1 × 10^4^, 1 × 10^5^, or 1 × 10^6^ CSC-hGC^[GFP+LUC]^ in 100 *μ*l of PBS at the lower midline into each side of the abdominal cavity. When 1 × 10^6^ CSC-hGC^[GFP+LUC]^ were injected, tumors were formed from the intraperitoneally transplanted cells in the abdominal cavity of each mouse (Figure 1(d)). Thus, a dose of 1 × 10^6^ CSC-hGC^[GFP+LUC]^ was adopted to establish the peritoneal metastasis model in our study. After validation of this mouse model two times via in vivo bioluminescence imaging, subsequent experiments based on the mouse model were performed according to the same procedure. Before the second intraperitoneal transplantation, the first-generation pMCSC-tGC (pMCSC-tGC^[G1]^) were passaged for 3-4 generations.

### 2.4. In Vivo Bioluminescence Imaging

Mice were sedated with 4% chloral hydrate (400 mg/kg) and given a single intraperitoneal (i.p.) 150 mg/kg dose of D-luciferin (Nanjing Asian Chemical Co., Ltd., China) in PBS. Bioluminescence imaging with a CCD camera (Xenogen, U.S.) was initiated 10 minutes after injection of D-luciferin. The imaging time ranged from 1 to 60 seconds according to the amount of luciferase activity.

Bioluminescence was assessed in the region of interest (ROI), which was defined manually. All data are expressed in units of photon flux (photons/s/cm^2^/steradian) and were collected and analyzed using the in vivo imaging system (Xenogen, U.S.).

### 2.5. Spherical Culture of pMCSC-tGC

Four weeks later, all mice were sacrificed by CO_2_ inhalation. The transplanted GC tumors were harvested and washed 5 times with PBS containing 10% penicillin-streptomycin solution (HyClone, U.S.). Then, to obtain a single-cell suspension, the samples were cut into pieces and digested with collagenase IV in a 37°C incubator for 30-60 minutes based on their size. Cell suspensions were collected after filtering through a 70 *μ*m filter. After centrifugation at 1000 r/min for 5 minutes, the cell pellet was collected and cultured in serum-free DME/F12 medium in a CO_2_ incubator at 37°C. The cells isolated from the first generation of transplanted tumors derived from CSC-hGC in serum-free culture medium were defined as pMCSC-tGC^[G1]^. Similarly, pMCSC-tGC^[G1]^ were intraperitoneally injected to establish the second generation of transplanted tumors. Cell spheres isolated from the transplanted tumors derived from pMCSC-tGC^[G1]^ were defined as second-generation pMCSCs (pMCSC-tGC^[G2]^).

### 2.6. Morphological Assessment of Transplanted Tumors

The intraperitoneally transplanted tumors derived from CSC-hGC, pMCSC-tGC^[G1]^, and pMCSC-tGC^[G2]^ were excised by laparotomy, fixed with 10% buffered formalin, embedded in paraffin, sliced, and stained with hematoxylin-eosin (H&E) (ZSGB-BIO, Beijing, China; Sigma, U.S.) before microscopic observation. Additionally, frozen sections were prepared from fresh tissues of transplanted tumors derived from CSC-hGC^[GFP-LUC]^, and the sections were then stained with DAPI (Sigma, U.S.). Fluorescence microscopy (Olympus, Japan) was used to visualize tumor cells.

### 2.7. Immunohistochemistry (IHC)

The expression levels of CD44, CD54, E-cadherin (E-cad), *α* smooth muscle actin (*α*-SMA), and Snail were examined in the two generations of transplanted tumors by IHC. The reagents used for immunohistochemical staining included a primary mouse anti-human E-cad monoclonal antibody (Abcam, Hong Kong, China), a primary rabbit anti-human *α*-SMA monoclonal antibody (ProMab Biotechnologies, Inc., U.S.), a primary rabbit anti-human CD44 monoclonal antibody (eBioscience, U.S.), a primary rabbit anti-human Snail polyclonal antibody (Abcam, Hong Kong, China), a primary rabbit anti-human CD54 monoclonal antibody (Abcam, Hong Kong, China), and secondary goat anti-mouse and anti-rabbit antibodies (Life Technologies, U.S.). Two independent pathologists assessed the immunohistochemical staining, and multiple fields on each slide were selected for microscopic evaluation of the staining intensity and extent in each group. Conflicting assessments were resolved by discussion and agreement.

### 2.8. Immunocytofluorescence (ICF)

Before ICF, cell spheres were digested into single cells with Accutase (Sigma, U.S.) at 37°C for 10 minutes and were then fixed with 4% paraformaldehyde, permeabilized with 0.5% Triton X-100, and stained with primary and secondary antibodies. First, cell slides were used for ICF, and frozen sections of cellular spheres were used in subsequent experiments, because the fluorescence of individual cells was too weak. For preparation of frozen sections, cell spheres were collected and washed with PBS three times. Then, the cell spheres were dehydrated with a 30% sucrose water solution for 18 hours. The sucrose water solution was discarded, and 200 ml of Tissue OCT-Freeze Medium (Solarbio, U.S.) was mixed gently with the cell spheres at room temperature. The suspension was solidified in a -20°C freezer and was then sliced into frozen sections. The frozen sections were stained with primary and secondary antibodies. Although different methods were used, the difference had a minimal effect on our measurements of relative protein expression levels. The expression of epithelial-mesenchymal transition- (EMT-) related markers and regulators, including E-cad, *α*-SMA, matrix metalloproteinase 2 (MMP2), matrix metalloproteinase 9 (MMP9), Survivin, Vimentin, ZEB1, OVOL2, and GRHL2, in CSC-hGC, pMCSC-tGC^[G1]^, and pMCSC-tGC^[G2]^ was evaluated by ICF. The reagents used for ICF included primary mouse anti-human E-cad monoclonal antibodies (BD Biosciences, U.S.), primary rabbit anti-human MMP2 polyclonal antibodies (Abcam, Hong Kong, China), primary rabbit anti-human MMP9 monoclonal antibodies (Abcam, Hong Kong, China), a primary rabbit anti-human *α*-SMA monoclonal antibody (Abcam, Hong Kong, China), a primary rabbit anti-human Survivin monoclonal antibody (Abcam, Hong Kong, China), a primary rabbit anti-human Vimentin monoclonal antibody (Cell Signaling Technology, U.S.), a primary mouse anti-human OVOL2 monoclonal antibody (Santa Cruz Biotechnology, U.S.), primary rabbit anti-human ZEB1 monoclonal antibodies (Abcam, Hong Kong, China), primary rabbit anti-human anti-GRHL2 polyclonal antibodies (Sigma-Aldrich, U.S.), and secondary goat anti-mouse and rabbit antibodies (Life Technologies, U.S.). Multiple fields were selected for microscopic evaluation of the staining intensity and extent in each group. The same methods were used for the 3 groups of samples.

### 2.9. Quantitative Real-Time PCR (q-PCR)

Total RNA was extracted with TRIzol (Life Technologies, U.S.) from CSC-hGC, pMCSC-tGC^[G1]^, and pMCSC-tGC^[G2]^. A TAKARA kit (TaKaRa Biotechnology Co., Ltd., Dalian, China) was used for quantitative real-time PCR. The 10 *μ*l reaction volume contained 5 *μ*l of SYBR premix Ex Taq™, 0.8 *μ*l of DNA template, 0.4 *μ*l of each primer, and 3.4 *μ*l of dH_2_O. The thermal cycling conditions used for PCR were as follows: 95°C for 30 seconds and 40 cycles of 5 seconds at 95°C and 30 seconds at 60°C. GAPDH was used as the internal control. All samples were measured in triplicate, and the PCR data were analyzed using the 2^-*ΔΔ*Cq^ method. The same methods were used for the 3 groups of samples.

### 2.10. Transwell Assay

Before the transwell assay, cell spheres were digested into single cells with Accutase (Sigma, U.S.) at 37°C for 10 minutes, and the cells were then suspended in serum-free medium. A 24-well plate with transwell cell culture chambers (Corning, U.S.) was used. To acclimate the transwell cell culture chambers, 100 *μ*l of serum-free DME/F12 medium (HyClone, U.S.) and 600 *μ*l of DMEM (HyClone, U.S.) supplemented with 10% fetal bovine serum (Gemini, U.S.) were added to the upper and lower chambers, respectively. Twelve hours later, the medium was removed from both chambers. Cells (5 × 10^4^ cells/well) were suspended in 100 *μ*l of serum-free medium and seeded in the upper chamber. In addition, 600 *μ*l of DMEM supplemented with 10% fetal bovine serum was added to the lower chamber. The cells remaining on the upper surface of the filter in the upper chamber after incubation for 18 hours at 37°C in a CO_2_ incubator were removed by wiping. The remaining cells were fixed and stained with Wright Giemsa Solution (Jian Cheng Technology Co., Ltd., Nanjing, China). Cell migration was quantified by counting the cells that migrated to the lower surface of the filter. Cells in nine fields per filter were counted under a light microscope at a magnification of ×400. Most steps of the invasion experiment were similar to those described above for the migration experiment, except that Matrigel Matrix (BD Biosciences, U.S.) diluted with DME/F12 medium (1 : 5) was added to the upper chamber, and 1 × 10^5^ cells/well were seeded. The same methods were used for the 3 groups of samples.

### 2.11. Statistical Analysis

Statistical analyses were performed using the SPSS software package (version 19.0; SPSS Inc., Chicago, IL, U.S.). Continuous variables are presented as the means ± standard deviations (SDs), and independent *t*-tests were used for comparisons. A probability (*p*) value of less than 0.5 was considered to indicate a statistically significant difference. All *p* values are two-sided.

## 3. Results

### 3.1. Establishment of the Mouse Model of Peritoneal Metastasis by CSC-hGC

**Figure 2:**
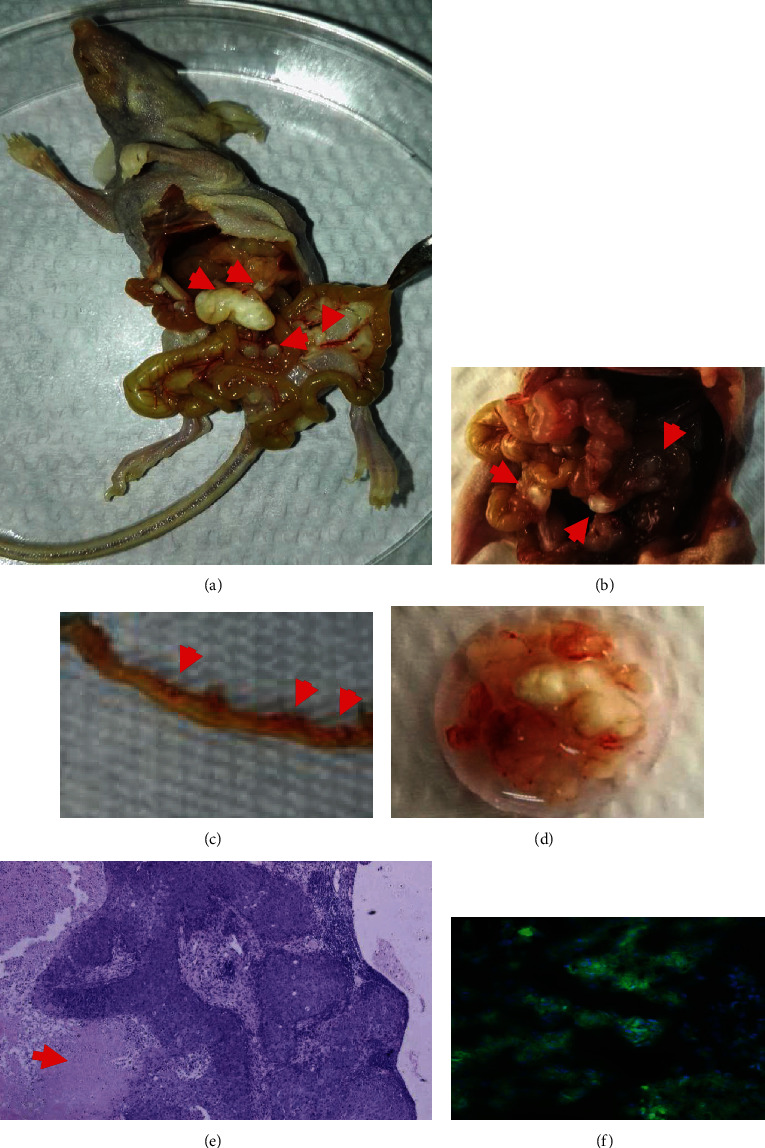
(a, b) In the sequential transplantation, gross anatomy generally showed that transplanted tumors, oval-shaped, with diverse size up to maximal 0.6 cm, were located at the greater omentum and interintestinal space. (c) Those tumors distributed along mesenteric vessel with grain-like appearance. (d) When dissected, the tumor tissue presented creamy white color, irregular shape, and hard texture. (e) Histology by H&E staining (×100) showed that tumor cells were clustered, and nuclei were large and with mitotic appearance. Necrotic regions were observed around tumor cell clusters, and no glandular structure was observed in tumor tissue with poor differentiation. (f) The frozen section under fluorescence microscope (×200) showed the formation of intraperitoneal transplanted tumor from the CSC-hGC^[GFP+LUC]^ (green: tumor cells; blue: DAPI staining for nuclei).

CSC-hGC^[GFP+LUC]^ with stable expression of GFP+LUC were successfully generated (Figures 1(b)–1(d)). Animal experiments were completed with the designed sample size without adverse events other than tumorigenesis. Four weeks after intraperitoneal injection of CSC-hGC^[GFP+LUC]^, the mouse model of GC peritoneal metastasis was stably established with 1 × 10^6^ injected cells (Figure 1(d)). CSC-hGC^[GFP+LUC]^ formed intraperitoneal tumors, as shown by H&E staining (Figures 2(a)–2(e)). Additionally, fluorescence microscopy of frozen sections showed that tumor cells emitted green fluorescence (Figure 2(f)) and validated that the peritoneal tumors originated from CSC-hGC^[GFP+LUC]^. The subsequent experiments were performed effectively with that number of cells.

### 3.2. Stemness and Tumorigenicity of pMCSC-tGC

**Figure 3:**
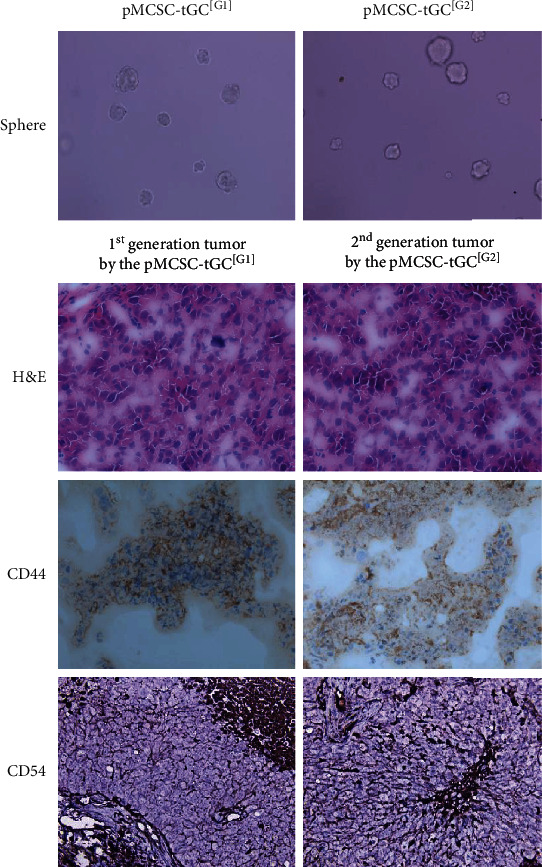
The tumor spheres (×200) of the pMCSC-tGC^[G1]^ and pMCSC-tGC^[G2]^ and correspondingly the H&E staining (×200) and immunohistochemistry of CD44 and CD54 (×200) for their intraperitoneally transplanted tumors.

pMCSC-tGC^[G1]^ (id: pMCSC.112.p1) and pMCSC-tGC^[G2]^ (id: pMCSC.112.p2) were successfully isolated through spherical culture of the transplanted tumor cells (Figure 3). Both pMCSC-tGC^[G1]^ and pMCSC-tGC^[G2]^ formed dispersed peritoneal tumors after intraperitoneal injection. H&E staining histologically confirmed the tumorigenicity of pMCSC-tGC^[G1]^ and pMCSC-tGC^[G2]^ and demonstrated the similarly poor differentiation of transplanted tumors derived from CSC-hGC, pMCSC-tGC^[G1]^, and pMCSC-tGC^[G2]^ (Figure 3). In addition, the putative membrane markers of CSC-hGC, CD44 and CD54, were expressed in the first and second generations of transplanted tumors (Figure 3).

### 3.3. Phenotypes of Mesenchymal-Epithelial Transition (MET) of pMCSC-tGC

**Figure 4:**
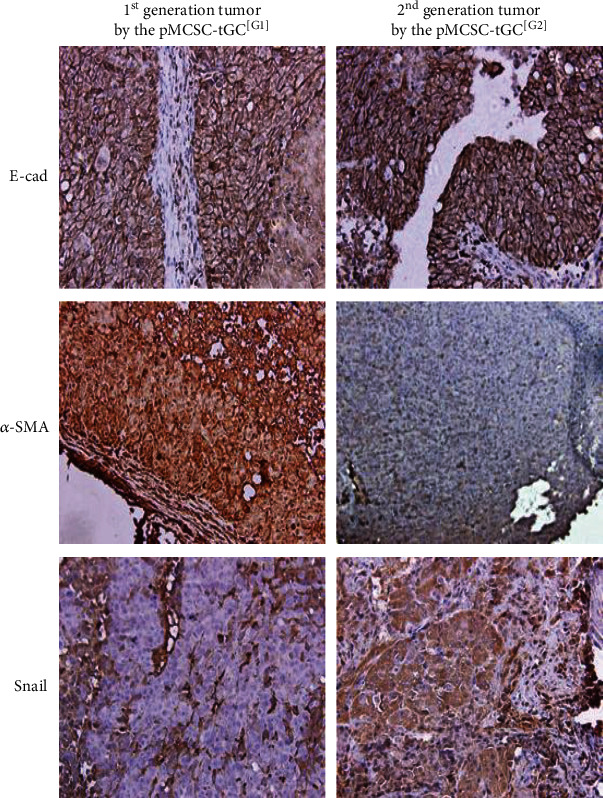
Immunohistochemistry of E-cad, *α*-SMA, and Snail (×200) for the two generations of intraperitoneally transplanted tumors from the pMCSC-tGC^[G1]^ and pMCSC-tGC^[G2]^.

**Figure 5:**
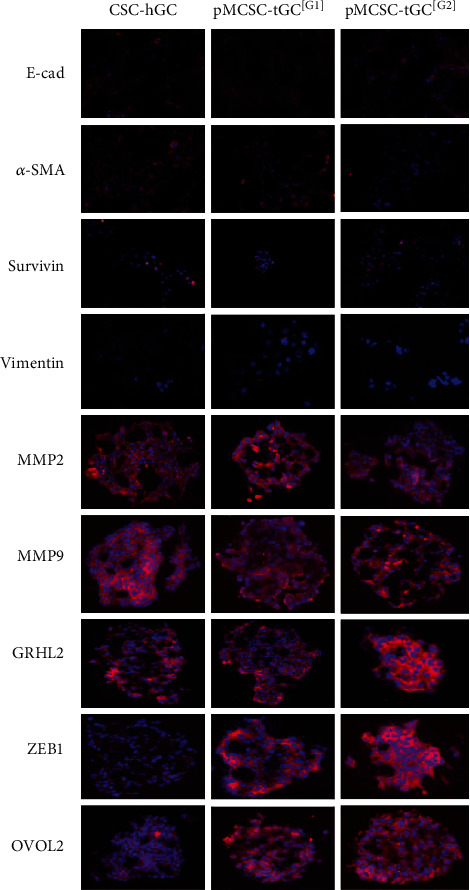
Immunocytofluorescence of E-cad, *α*-SMA, Survivin, Vimentin, MMP2, MMP9, GRHL2, ZEB1, and OVOL2 (×400) for the CSC-hGC, pMCSC-tGC^[G1]^, and pMCSC-tGC^[G2]^ cells.

**Figure 6:**
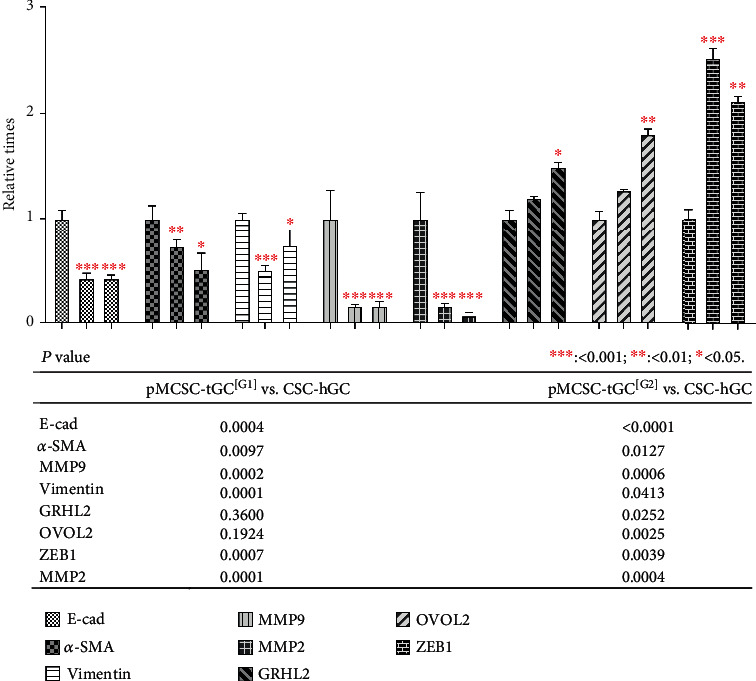
Quantitative real-time PCR of E-cad, *α*-SMA, MMP9, Vimentin, GRHL2, OVOL2, ZEB1, and MMP2 for the CSC-hGC, pMCSC-tGC^[G1]^, and pMCSC-tGC^[G2]^ cells.

E-cad and Snail (evaluated in vivo to assess the homing status) were upregulated but *α*-SMA was downregulated in the transplanted tumors derived from pMCSC-tGC^[G2]^ compared with the transplanted tumors derived from pMCSC-tGC^[G1]^ (Figure 4). The ICF results showed that after sequential formation of intraperitoneal tumors, E-cad, Survivin, Vimentin, MMP2, and MMP9 (evaluated in vitro to assess the cell status) exhibited stable expression in pMCSC-tGC cells, *α*-SMA exhibited decreased expression. However, OVOL2, GRHL2, and ZEB1 exhibited increased expression in pMCSC-tGC^[G2]^ (Figure 5). RT-PCR of pMCSC-tGC cells (in vitro) revealed that compared with the corresponding levels in CSC-hGC, the expression levels of mRNAs encoding E-cad (*p* < 0.0001), MMP9 (*p* = 0.0006), *α*-SMA (*p* = 0.0127), Vimentin (*p* = 0.0413), and MMP2 (*p* = 0.0004) were decreased in pMCSC-tGC^[G2]^, while the expression levels of mRNAs encoding ZEB1 (*p* = 0.0039), OVOL2 (*p* = 0.0025), and RGHL2 (*p* = 0.0252) were increased in pMCSC-tGC^[G2]^ (Figure 6).

### 3.4. Enhanced Migration and Invasion of pMCSC-tGC

**Figure 7:**
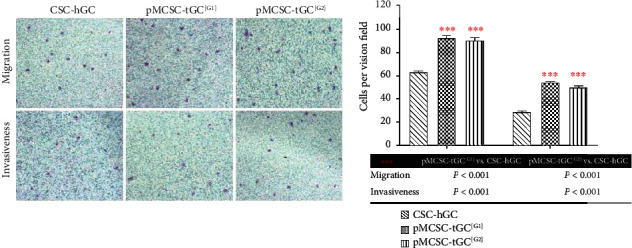
Transwell assays (×400) for the cell mobility of the CSC-hGC, pMCSC-tGC^[G1]^, and pMCSC-tGC^[G2]^.

The transwell assay results demonstrated that the numbers of migrated (92.3 ± 2.5 vs. 62.0 ± 2.0, *p* < 0.001) and invaded (54.7 ± 1.2 vs. 28.7 ± 1.2, *p* < 0.001) pMCSC-tGC^[G1]^ were higher than those of CSC-hGC. Similarly, the numbers of migrated (91.0 ± 2.6 vs. 62.0 ± 2.0, *p* < 0.001) and invaded (52.7 ± 2.1 vs. 28.7 ± 1.2, *p* < 0.001) pMCSC-tGC^[G2]^ were higher than those of CSC-hGC (Figure 7).

## 4. Discussion

In this preliminary study, we proposed a novel hypothesis regarding the mechanism underlying peritoneal metastasis of GC, postulating that it is derived from a potential cluster of pMCSCs. To our knowledge, this study is the first to investigate the effect of pMCSCs on peritoneal metastasis. We also successfully established a nude mouse model of peritoneal metastasis through intraperitoneal injection of CSC-hGC. In this animal model, we performed sequential intraperitoneal transplantation and isolated the first- and second-generation pMCSC-tGC. The stemness and tumorigenicity of pMCSC-tGC^[G1]^ and pMCSC-tGC^[G2]^ well succeeded to the CSC-hGC. Sequential intraperitoneal transplantation possibly enhanced the partial epithelial phenotypes of pMCSC-tGC and strengthened their migratory and invasive capabilities.

Researchers have isolated gastric CSCs from various GC cell lines and from tumor tissues and peripheral blood of GC patients. Different CSC markers, such as Lgr5, CD44, CD133, CD54, DLL4, and CD24, have been reported [23–26]. However, the specific CSC markers are still controversial and under discussion. In our previous study, gastric CSCs were identified and isolated from tumor tissues and peripheral blood of gastric adenocarcinoma patients through the expression of the CSC markers CD44 and CD54 [18]. Our study indicated that the cells with a CD44(+)CD54(+) phenotype had characteristics of stem cells. First, those cells had the capabilities for sphere formation and self-renewal in serum-free medium, while in medium containing serum, the number of cells with the CD44(+)CD54(+) phenotype was decreased. Second, the results of animal experiments indicated that these cells had strong tumorigenic properties and could form tumors continuously. In addition, subcutaneous xenografts derived from those cells in nude mice exhibited histologic features similar to those of the primary GC tissue. Compared with GC cell lines, those cells had stronger invasion, metastasis, self-renewal, and proliferation abilities. Thus, we believed that the clusters of CD44(+)CD45(+) cells isolated from tumor tissues and peripheral blood of gastric adenocarcinoma patients were gastric CSCs.

Previous studies have investigated the mechanism underlying peritoneal metastasis of GC in vitro [27, 28]. Several models of peritoneal metastasis have been established by orthotopic implantation techniques and intraperitoneal injection of human GC cell lines [29–33]. We established a nude mouse model via intraperitoneal injection of CSC-hGC. Through in vivo bioluminescence or fluorescence imaging, gene and cell marker expression can be observed in vivo in living animals [34, 35]. GFP and LUC have already been widely used in this system for evaluating single cells in vitro and for functional testing in vivo [36–39]. In this study, we monitored and validated the tumorigenicity of CSCs and pMCSCs via in vivo bioluminescence imaging.

CSCs have been implicated not only in initiating and sustaining primary tumor growth but also in driving the seeding and formation of metastases at distal sites [40–44]. The existence of the MCSC population has been demonstrated in various human cancers. For example, both CD133(+)CXCR4(+) and CD133(+)CXCR4(-) pancreatic CSCs can form tumors, but only the CD133(+)CXCR4(+) subset has migration capability [45]. In colorectal cancer, the metastatic phenotype is determined by a distinct subpopulation of CD26(+) CSCs [46]. Associations between MCSCs and metastasis have also been found in several types of carcinomas [45–47]. In this study, interestingly, the identified pMCSC-tGC initially exhibited or acquired enhanced migratory and invasive capabilities after intraperitoneal transplantation. The Ras-MAPK signaling pathway is involved in tumor cell migration and invasion by regulating ECM degradation and mediating cell adhesion and movement [48, 49]. However, whether the Ras-MARK signaling pathway was activated under the conditions established in this study is unclear, and this possibility requires further study and discussion. pMCSCs can be enriched from the CSC-hGC population through this animal model, implying that peritoneal metastasis of GC may be associated with clusters of pMCSCs.

EMT is often activated during cancer invasion and metastasis and is characterized by loss of epithelial differentiation and a shift towards a mesenchymal phenotype [50, 51]. EMT is potentially associated with a set of markers, such as members of the cadherin family and *α*-SMA. E-cad, an epithelial marker, is the most commonly investigated marker and has been found to be strongly associated with the EMT process by maintaining the stability of intercellular junctions. Expression of E-cad negatively correlates with EMT and cell mobility. Another member of the cadherin family, P-cad, acts as a double-edged sword, as either a tumor suppressor or oncogenic protein [52]. Moreover, P-cad overexpression is associated with cancer cell invasion and metastatic dissemination [52]. However, the functional role of P-cad was proven to be dependent on the cellular context of E-cad in a breast cancer model [53]. Therefore, in the present study, only E-cad and not P-cad was evaluated to assess the tendency towards EMT.

The results of IHC (evaluated in in vivo tumors to assess the homing status) indicated that the expression of E-cad in pMCSC-tGC^[G2]^ was upregulated, but the ICF results (evaluated in vitro in free cells to assess the migration status) indicated that E-cad expression in these cells was stable, suggesting an epithelial phenotype indicating a tendency towards MET. However, the consistent phenotype of *α*-SMA downregulation in pMCSC-tGC^[G2]^ observed in the immunohistochemical, ICF, and RT-PCR assays indicated the tendency towards MET, as did the downregulation of Vimentin. The transition from an epithelial to a mesenchymal phenotype involves downregulation of epithelial markers such as E-cad and upregulation of mesenchymal markers such as Vimentin and *α*-SMA [54–56]. Therefore, our major findings suggested that pMCSC-tGC^[G2]^ potentially exhibited an enhanced epithelial phenotype, namely, a tendency towards a transition from a mesenchymal to an epithelial phenotype.

The key molecular markers of pMCSC-tGC, E-cad and *α*-SMA, were evaluated under different growth conditions. We hypothesized that in vitro free cells (migration-mesenchymal phenotype) and in vivo tumors (homing-epithelial phenotype) might exhibit differential expression of EMT markers. Our results generally supported this hypothesis. Two generations of cells, pMCSC-tGC^[G1]^ and pMCSC-tGC^[G2]^, exhibited stable or downregulated expression of E-cad as in vitro free cells (mesenchymal status) but upregulated expression of E-cad as in vivo tumor cells (epithelial status). Similarly, *α*-SMA, a mesenchymal marker, was downregulated in in vivo tumor cells (epithelial status), consistent with the upregulation of E-cad.

Additionally, members of the MMP family can induce EMT and promote tumor cell mobilization by degrading the basement membrane and destroying intercellular junctions [54, 57]. In our study, the expression of MMP2 and MMP9 was downregulated, as determined by PCR. These findings also indicated that MET might occur during homing. Finally, not only is Snail a key inducer of EMT, but it also has EMT-independent functions in cell survival and stem cell biology [58]. The upregulation of Snail observed here may be inferred to be a function of stemness maintenance rather than EMT.

Recently, MET has been observed in metastases of most human cancers [59–61]. The progression of solid tumors is accompanied by EMT, through which tumor cells acquire enhanced invasive and metastatic capabilities. In the appropriate microenvironments of target organs, disseminated tumor cells must undergo the inverse transition, i.e., MET, for successful metastasis. Initiation of tumor growth at the secondary site has been proposed to be a dynamic process, and both EMT and MET have been indicated to be the driving forces of metastasis in different stages [51, 61, 62]. Chaffer et al. demonstrated the importance of the epithelial phenotype in secondary tumor formation by a cell line from human transitional carcinoma of the bladder [63]. In addition, previous studies showed that MET is necessary for the colonization and metastasis of differentiated cancers and that cancer cell growth is arrested if EMT is not reversed to MET [64, 65].

EMT/MET has long been viewed primarily as a binary process [66–68]. However, recent studies have shown that during the transition between the epithelial (E) and mesenchymal (M) phenotypes, cells can acquire a stable hybrid epithelial/mesenchymal (E/M) phenotype or a partial EMT phenotype. These hybrid E/M cells that coexpress epithelial and mesenchymal markers have mixed epithelial and mesenchymal properties [69, 70]. The hybrid E/M phenotype is related to the stemness of cancer cells, and CSCs in several cancers (such as breast, prostate, lung, and colorectal cancers) have been found to coexpress epithelial markers and mesenchymal markers, suggesting a hybrid E/M phenotype [71–76]. In the present study, we found that epithelial markers and mesenchymal markers were expressed simultaneously in CSC-hGC, as well as in pMCSC-tGC^[G1]^ and pMCSC-tGC^[G2]^, suggesting that all of these cells had a hybrid E/M phenotype. In addition, compared to CSCs, both pMCSC-tGC^[G1]^ and pMCSC-tGC^[G2]^ showed a decreasing trend in the levels of epithelial and mesenchymal markers as measured by quantitative real-time PCR, indicating that pMCSC-tGC were more likely to be hybrid E/M ones. Previous studies reported that compared to the pure M or E phenotype, the hybrid E/M phenotype and the partial E/M phenotype are correlated with increased invasive and metastatic potential [69–72]. Our results showed that pMCSC-tGC^[G1]^ and pMCSC-tGC^[G2]^ acquired enhanced migratory and invasive capabilities, further showing that pMCSC-tGC might acquire a hybrid E/M phenotype. EMT can be induced by overexpression of EMT-related transcription factors (termed EMT-TFs), such as Snail and ZEB1/2 [77–79], while MET can also be induced by a set of MET-TFs such as OVOL1/2 and RGHL2 [78–80]. Recent studies demonstrated that ZEB1, GRHL2, and OVOL2 are components of the core EMT regulatory circuit and play important roles in regulating EMT dynamics and stabilizing the E, M, and E/M phenotypes [70, 76–80]. Complete MET has been reported to require overexpression of MET-TFs such as GRHL2 and/or OVOL2 and knockdown of the EMT-TFs such as ZEB1 [70, 78]. To further investigate the complete or partial MET of pMCSC-tGC, we measured the changes in the expression of ZEB1, OVOL2, and GRHL2 in CSC-hGC, pMCSC-tGC^[G1]^, and pMCSC-tGC^[G2]^. ZEB1, GRHL2, and OVOL2 were upregulated in pMCSC-tGC, and we considered that GRHL2 and OVOL2 probably promote MET, whereas ZEB1 might inhibit MET during peritoneal metastasis of CSC-hGC, which also suggested that pMCSC-tGC exhibit partial MET [70, 78]. EMT and MET can be regulated by numerous other mechanisms in addition to transcriptional control mechanisms, such as long noncoding RNAs, microRNAs, epigenetic modifications, alternative splicing, and posttranslational alterations in protein stability [81–87]. These mechanisms can differ among cell types or tissues of origin [76]. Our preliminary study showed that homing to the peritoneum through partial MET was necessary for pMCSC-tGC, and this finding requires further investigation.

In conclusion, a mouse model of GC peritoneal metastasis based on CSC-hGC transplantation was established, and pMCSC-tGC were isolated by sequential intraperitoneal transplantation. This strategy is a novel approach for investigating and understanding the potential mechanism of GC peritoneal metastasis. Compared to CSC-hGC, pMCSC-tGC had an enhanced invasive capability, possibly due to partial MET during homing and the formation of peritoneal colonies. pMCSCs may be the driving force underlying peritoneal metastasis of GC. Further investigations are needed to gain a clearer understanding of the complex process of the EMT-MET shift during peritoneal metastasis, and these preliminary findings require validation in further experiments.

## Data Availability

The data can be available by the email chenxinzu@scu.edu.cn.

## Additional Points

*Core Tip*. Cancer stem cells (CSCs) of human gastric cancer (GC) (termed CSC-hGC) were previously isolated. The nude mouse model of intraperitoneally transplanted CSC-hGC was successful in establishing sequential formation of peritoneal tumors and enrichment of pMCSCs. pMCSCs might be a specific subpopulation that can be sequentially enriched in an intraperitoneal transplantation model. pMCSCs exhibited a tendency towards partial mesenchymal-epithelial transition, enhancing their invasiveness during homing and the formation of peritoneal tumors. However, these preliminary findings require validation in further experiments.
